# Upon the Decomposition of Some Vegetable or Animal Substances, Submitted to the Action of Heat

**Published:** 1811-06

**Authors:** M. Gay-Lusac


*Upon the Decomposition of some Vegetable or Animal Substances, submitted to the Action of Heat. By* M. GAY-LUSAC. Communicated to the Society D'Arcueil, November, 1809.

WHEN we submit to distillation certain substances of a vegetable or animal nature, such as oxalic acid, indigo, &c. one portion of them is decomposed, and another is volatilized without experiencing any alteration. The proof that these effects are not owing to the impurity of these substances is, that if the portion which has been volatilized be again distilled, it is again decomposed proportionally, as much as at the first time; so that by frequently repeating the operation, we obtain a complete decomposition.

These facts, though very curious, have not fixed the attention of chemists. I shall endeavour to explain them on the principles which I have established in a Memoir on the Volatilization of Bodies, in the first vol. of the Society D'Arcueil. The question to resolve is, why, when we distil certain substances of a vegetable or animal nature, is one part of them decomposed, whilst the other part is volatilized? Why are they not wholly decomposed, or wholly volatilized?

The substances which present this kind of change are volatile, and at the same time susceptible of being decomposed by heat. Besides, a body cannot be volatilized before the period when its vapours have an elastic power sufficiently great to overcome the weight of the atmosphere, unless they can mix with the air, or with any other elastic fluid.

Now, if we submit to the action of heat a volatile substance capable of being decomposed, it may happen, either that it will be completely volatilized before it has acquired a temperature sufficient to decompose it, or that it will be decomposed before its vapours have attained elasticity enough to overcome the pressure of the atmosphere.

The first case presents no difficulty; it is that of the distillation of acetic acid, of alcohol, of ether, of the volatile oils, &c. The substances comprized in the second, such as indigo, the gallic, oxalic, and succinic acids; wax, tallow, the fixed oils, &c. will suffer decomposition before they are volatilized; but their decomposition gives rise to gases, and these gases will determine the volatilization of the undecomposed part, in the same manner as the air determines that of water below the temperature of its ebullition.

Since it is the gases resulting from the decomposition of a substance, which determine its volatilization, and prevent its complete complete destruction, and since all elastic fluids, in this respect, have similar properties, it is easy to volatilize indigo, several vegetable acids, and a number of other bodies, without occasioning them to undergo any alteration. It is sufficient to raise their temperature a little above that at which they are decomposed, and to make them pass through a current of an elastic fluid, which, in this circumstance, does not act upon them chemically.

These observations no doubt will receive frequent applications; it is from not having known them, that M. Chevreul has given an unsatisfactory explanation of the action of caloric upon indigo.

